# Toward Sustainable Adoption of Integrated Care for Prevention of Unplanned Hospitalizations: A Qualitative Analysis

**DOI:** 10.5334/ijic.7724

**Published:** 2024-06-28

**Authors:** Carmen Herranz, Alba Gómez, Carme Hernández, Rubèn González-Colom, Joan Carles Contel, Isaac Cano, Jordi Piera-Jiménez, Josep Roca

**Affiliations:** 1 Consorci d’AtencióPrimaria de Salut Barcelona Esquerra (CAPSBE), Barcelona, ES; 2 Fundacióde Recerca Clínic Barcelona - Institut d’Investigacions Biomèdiques August Pi i Sunyer (FRCB-IDIBAPS), Barcelona, ES; 3 Research and Innovation Management, Hospital Clínic de Barcelona, Barcelona ES; 4 Centro de Investigación Biomédica en Red de Enfermedades Respiratorias (CIBERES), Madrid, ES; 5 Chronic Care Program, Ministry of Health of Government of Catalonia, Barcelona, ES; 6 Universitat de Barcelona (UB), Barcelona, ES; 7 Digitalization for the Sustainability of the Healthcare System DS3-IDIBELL, L’Hospitalet de Llobregat, ES; 8 Faculty of Informatics, Multimedia and Telecommunications, Universitat Oberta de Catalunya, Barcelona, ES; 9 Catalan Health Service (CatSalut), Barcelona, ES; 10 Hospital Clínic de Barcelona (HCB), Barcelona, ES

**Keywords:** adaptive case management, design thinking, digitalization, personalized care, prevention of unplanned hospital admissions, Gestió adaptativa de casos, “Design Thinking”, Digitalització, Atenció personalitzada, Prevenció d’ingressos hospitalaris no planificats

## Abstract

**Introduction:**

Complex chronic patients are prone to unplanned hospitalizations leading to a high burden on healthcare systems. To date, interventions to prevent unplanned admissions show inconclusive results. We report a qualitative analysis performed into the EU initiative JADECARE (2020–2023) to design a digitally enabled integrated care program aiming at preventing unplanned hospitalizations.

**Methods:**

A two-phase process with four design thinking (DT) sessions was conducted to analyse the management of complex chronic patients in the region of Catalonia (ES). In Phase I, Discovery, two DT sessions, October 2021 and February 2022, were done using as background information: i) the results of twenty structured interviews (five patients and fifteen professionals), ii) two governmental documents on regional deployment of integrated care and on the Catalan digital health strategy, respectively, and iii) the results of a cluster analysis of 761 hospitalizations. In Phase II, Confirmation, we examined the 30- and 90-day post-discharge periods of 49,604 hospitalizations as input for two additional DT sessions conducted in November and December 2022.

**Discussion:**

The qualitative analysis identified poor personalization of the interventions, the need for organizational changes, immature digitalization, and suboptimal services evaluation as main explanatory factors of the observed efficacy-effectiveness gap. Additionally, a program for prevention of unplanned hospitalizations, to be evaluated during the period 2024–2025, was generated.

**Conclusions:**

A digitally enabled adaptive case management approach to foster collaborative work and personalization of care, as well as organizational re-engineering, are endorsed for value-based prevention of unplanned hospitalizations.

## Abstract (ca)

**Introducció:**

Els pacients crònics complexos són propensos a hospitalitzacions no planificades que comporten una gran càrrega per els sistemes sanitaris arreu del mon. Les intervencions per prevenir ingressos no planificats mostren resultats poc concloents pel que fa a efectivitat. Aquest estudi descriu una anàlisi qualitativa realitzada, en el marc de l’Acció Conjunta Europea JADECARE (2020–2023), per dissenyar un programa d’atenció integrada, amb suport digital, que te per objectiu prevenir les hospitalitzacions no planificades.

**Mètodes:**

L’estudi va consistir en un procés de co-disseny efectuat a partir de l’anàlisi de la gestió de pacients crònics complexos en la demarcació de Catalunya (ES). Es va efectuar en dues fases amb un total de quatre sessions de “Design Thinking (DT)”. A la Fase I, Discovery, es van fer dues sessions de DT, Octubre de 2021 i Febrer de 2022, utilitzant com a informació de fons: i) Els resultats de vint entrevistes estructurades (cinc pacients i quinze professionals), ii) Dos documents governamentals sobre el desplegament regional de l’atenció integrada i sobre l’estratègia de salut digital, respectivament, i iii) Els resultats d’una anàlisi de clústers de 761 hospitalitzacions. A la Fase II, Confirmació, vam examinar els resultats als 30 i 90 dies post-alta hospitalària de 49.604 casos en a dues sessions de DT addicionals realitzades durant els mesos de Novembre i Desembre de 2022.

**Discussió:**

L’anàlisi qualitativa va identificar: i) Insuficient personalització de les intervencions, ii) Necessitat de canvis organitzatius, iii) Immaduresa de la digitalització, i iv) Avaluació sub-óptima del desplegament dels serveis com a principals factors explicatius de la bretxa entre eficàcia i efectivitat observada en estudis previs. L’estudi proposa un programa de prevenció d’hospitalitzacions no planificades, a avaluar durant el període 2024–2025.

**Conclusions:**

Els resultats de l’anàlisi qualitativa donen suport a la modalitat de gestió adaptativa de casos, amb suport digital, per fomentar el treball col·laboratiu i la personalització de l’atenció. També plantegen la necessitat de reenginyeria dels processos clínics per assolir una prevenció eficient i basada en valors, de les hospitalitzacions no planificades en el mon real.

## Introduction

Complex chronic patients, as defined in [1], frequently face unplanned hospitalizations, either due to episodes of exacerbation, or during periods associated with increased vulnerability, like the transition from hospital to the community after hospital discharge [2]. It is widely accepted that unplanned hospital admissions generate significant dysfunctions, and a high financial burden, on healthcare systems worldwide [3].

The reports from international organizations [4,5], as well as historical randomized controlled trials [6], indicate that a significant percentage, up to fifty percent, of unplanned hospitalizations are avoidable. However, the existing literature examining interventions to reduce admissions in selected complex chronic patients with episodes of severe exacerbations [7] and in transitional care programs [8] aiming at decreasing early readmissions after hospital discharge present inconclusive findings [9]. This can be attributed to the heterogeneity of the interventions and/or suboptimal description of the protocols leading to poor comparability, among other factors. Overall, there is a clear consensus on the need to explore the potential of clinical processes to efficiently prevent unplanned hospitalizations within a care continuum scenario [9].

The integrated care case practice explores the hypothesis that the identification of actionable factors determining adoption could facilitate the design of a care continuum program to efficiently reduce unplanned hospitalizations in selected complex chronic patients. To this end, the current qualitative analysis was undertaken within the Catalan stakeholders of the European Union (EU) Joint Action on Implementation of Digitally Enabled Integrated Person-Centered Care (JADECARE) [10], running in the period 2020–2023.

Briefly, JADECARE intended to reinforce the capacity of health authorities to successfully address important aspects of health system transformation. To achieve these goals, four “Early Adopters” of original Good Practices supported 21 regions, from 16 EU countries, that participated in the project as “Next Adopters”. The Catalan Open Innovation Health Care Hub for ICT-supported Integrated Care was one of the four original Good Practices. It included as core members: i) the Department of Health of the Catalan Government, through the Integrated Care Agency, ii) the single-public payer (CatSalut), and iii) the Integrated Health District of Barcelona-Esquerra (AISBE encompassing 520 k citizens).

Two factors triggered the current integrated care case practice. Firstly, during the pre-implementation phase of JADECARE, internal discussions within the Catalan Good Practice highlighted the necessity of reevaluating the preventive management of complex chronic patients. The second triggering factor was the unsolved dissociation between the results of previous randomized controlled trials (RCT) [6] and those seen in a real-world setting [11], that is, the efficacy-effectiveness gap [11] often observed in complex interventions [12].

The current study focused on designing and adopting person-centered integrated care interventions for selected complex chronic patients to prevent unplanned hospitalizations. This approach covered two situations: firstly, selected patients showing a high risk of severe exacerbations due to clinical severity, poor self-management and/or environmental factors; and secondly, transitional care strategies after hospital discharge.

Ultimately, the targeted objectives in this integrated care case practice are: i) To identify key factors explaining the observed efficacy-effectiveness gap; ii) To produce well-defined interventions, including identification of target candidates, as well as the steps needed to achieve mainstream adoption, and iii) To evaluate the potential for generalization/transferability of such interventions at the EU level.

The report sequentially describes the regional context of the analysis, the main achievements of the two-phase co-design process based on design thinking sessions, and the process for the definition of the program for the prevention of unplanned hospitalizations to be tested during a two-year period, 2024–2025.

## Development of the Integrated Care Case Practice

### Setting the scene

Since 1981, the Catalan Health System [13,14] has been fully responsible for policy formulation, organization management, service delivery, and financial aspects of health services in Catalonia (ES), with 7.7 million citizens. This regional system ensures universal coverage and free access at the point of service [15,16]. Basic principles, traits and outcomes of the regional health system are reported in [14,15,16,17,18]. Regional health planning [19,20] has played a backbone role in triggering the health system transformations toward digitally supported integrated health and social care services during 2011–2020. A myriad of interconnected service modalities currently available for chronic care management is outlined in Table 1.

**Table 1:** Service modalities supporting care continuum in Catalonia.


SERVICE MODALITIES	CHARACTERISTICS

**Primary Care**	

Primary Health Centers	Are the first point of contact for individuals seeking healthcare services and coordinate patients’ cure and/or care.

Home Care	Home-based support services directly provided by the primary care center

**Intermediate Care (socio-health services) – Outpatient regime**	

Specialized support teams	1. UFISS – Professionals (physician, nurse, and social worker) devoted to assessment of complex geriatric cases. Ascribed to intermediate care hospitals. 2. EAIA – Dedicated to detection and management of multimorbid patients with high social risk showing acute clinical episodes. 3. ETODA – Stands for outpatient direct observation therapy teams devoted to a specific program for tuberculosis therapy. The aim is to guarantee the correct performance of the treatment by patients with social problems, through the direct supervision of the administration of the medication.

Palliative care (PADES)	Interdisciplinary teams, coordinated with the primary care center, devoted to end-of-life care with a holistic approach.

Socio-health day hospitals	The objectives of day care services are assessment and comprehensive treatment, rehabilitation and ongoing maintenance care targeting geriatric or multimorbid patients.

**Intermediate Care (socio-health services) – Hospitalization**	

Long stay units	For rehabilitative treatment, maintenance care and prevention of complications, and as support for elderly people with long-term chronic diseases that have generated functional disabilities.

Convalescence units	Mid-stay unit to restore the functions or activities affected by health problems in geriatric multimorbid patients needing functional recovery after undergoing a surgical, medical, or traumatic process.

Sub-acute care units	For people with chronic and advanced disease who, due clinical exacerbation, need the continuation of a treatment under continuous clinical supervision. The aim of this care is to achieve clinical stabilization and comprehensive rehabilitation

Palliative care units	End-of-life hospitalizations

**Hospital Care – Hospital at Home**	

Full (Hospital Avoidance) or partial (Early Discharge) substitution of conventional in-patient admission by home hospitalization (administered by hospital-based professional teams) for patients showing clinical criteria of hospitalization due to an acute health event.	

**Social Support**	

Interdepartmental Plan of Social and Health Interaction approved in 2014. Further developed in [23]	


UFISS: Functional Unit for Socio-health care; EAIA: Integrated Care Team for specific target groups; ETODA: Team Directly Supporting Therapy on Outpatient basis.

Two distinct documents were used as background information in the development of the current integrated care case, each of them contributing to its conceptual framework. One document encompasses the fundamental characteristics of the regional integrated care model for chronic patients [1], providing operational definitions of the two profiles of candidates to the program for prevention of unplanned hospitalization: i) Complex chronic patients characterized by multimorbidity, > 65 years of age, and showing mild frailty, ii) Advanced chronic patients with a life expectancy of less than 18 months. These two groups correspond to the 5% (P_≥95_) of citizens at the tip of the population-based risk stratification pyramid at regional level.

The second report outlines the ongoing digital health strategies for 2021–2025 [22], specifically focusing on implementing a knowledge-based digital platform to support cloud-based health services. Moreover, the description of specific digital developments [21] providing support to collaborative adaptive case management across healthcare providers, currently used for peri-surgical care (prehabilitation), was also considered in the qualitative analysis.

### Setting the scene Co-design process: the methodological approach

**Figure 1:**
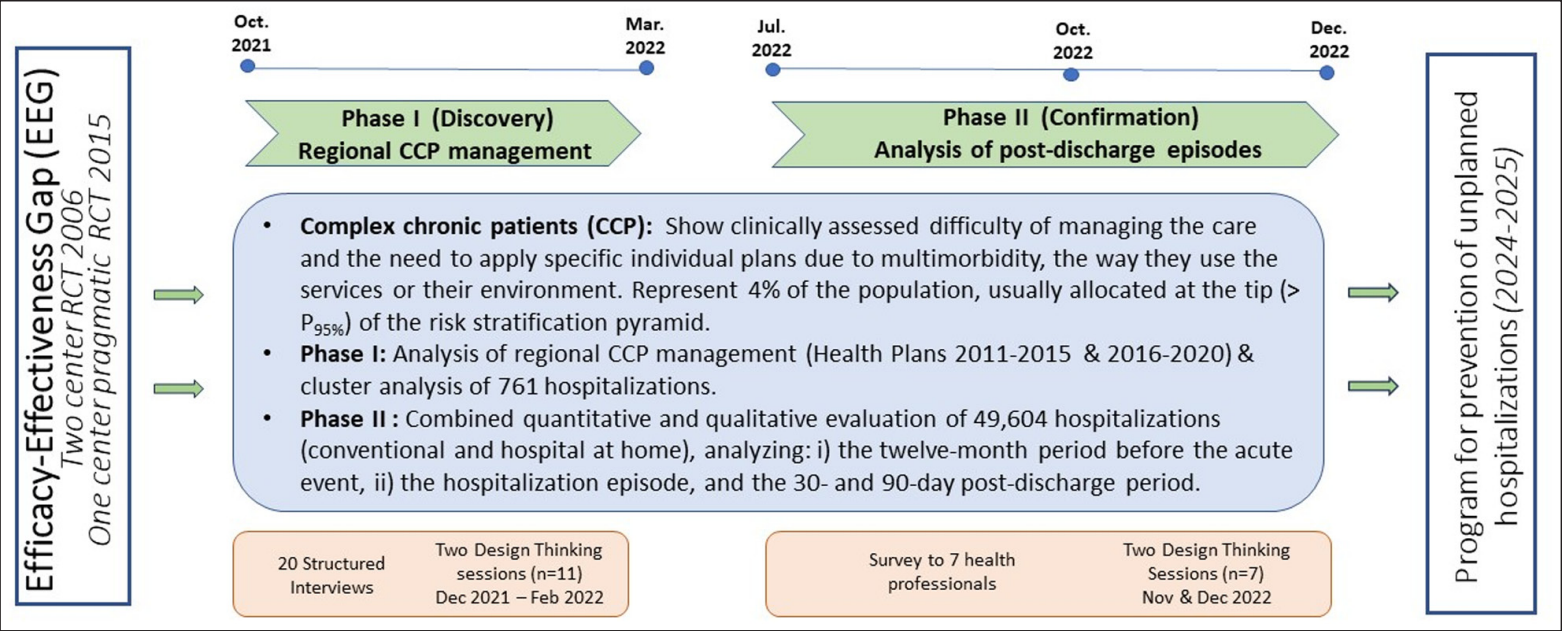
**Two-phase co-design process timeline – A trigger:** The efficacy-effectiveness gap (EEG) seen between two studies carried out in 2006 [6] and 2015 [12]. **The Discovery phase:** devoted to the analysis of regional Complex Chronic Patients (CCP) management and identifying the main explicable factors for the EEG. CCP represent 4% of the population, allocated above P95 of the population-based risk stratification pyramid. **The Confirmation phase**, assessing value generation of Hospital at Home, was used to analyse the interactions between the hospital teams and community-based services, reflecting the status of vertical integration [26]. **The final outcome** is the elaboration of a program for preventing unplanned hospitalizations to be tested during the period 2024–2025. DT: Design Thinking. RCT: Randomized controlled trial.

The qualitative analyses were conducted following a grounded theory approach [24], using a methodology like the one [25,26] described in detail in [27]. Figure 1 depicts the timeline and the focus of its two phases.

Phase I, entitled “Discovery”, had a twofold purpose: i) to identify actionable factors to enhance management of complex chronic patients; and ii) to generate specific recommendations to be tested during the period 2024–2025. The two design thinking sessions carried out in Phase I were done in December 2021 and February 2022, respectively.

Phase II, entitled “Confirmation”, aimed to consolidate the achievements of Phase I. The corresponding two design thinking sessions were conducted during the last quarter of 2022 [26].

Details on methodological aspects of the qualitative analysis are reported in the on-line supplementary material.

The Discovery phase – Between October to December 2021, in-depth interviews were conducted with 20 key informants. This group comprised 5 patients and 15 clinical professionals and healthcare service managers, chosen for their leadership in the Catalan Health System. The interviews, on average 45 minutes each, were carried out by the first author alongside a specialist in qualitative research and services design.

*Patients* – A total of 25 patients were selected from the community as potential candidates for the interviews. The inclusion criteria were: i) At least one hospital admission in the last year, ii) Two or more chronic pathologies and iii) Allocated > P_80_ in the Catalan risk stratification pyramid. We excluded those patients presenting: i) Difficulties in conducting an interview (sensory problems, cognitive impairments, or severe mental illness), and ii) Refusing to participate. The final five patients, with whom the interviews were conducted, were selected balancing the following criteria to achieve the highest representativeness: i) Male/female, ii) With/without caregiver and iii) With transition from hospital to home/hospital to intermediate care to home.

For the patients, the interviews were structured to capture the reported experience on the following aspects: i) initiation of the acute episode, ii) emergency room admission, iii) hospitalization period; iv) follow-up after hospital discharge, v) intermediate care, if needed, vi) home-based support and services, if needed; vii) primary care services; and viii) care continuum. All of them signed an approved informed consent form to participate in the interviews (NCT04056663).

Professionals **(Table 1S)** – Fifteen experts were selected based on their leadership in integrated care, as well as the coverage of three areas: i) Clinical activities (physicians, nurses, and social workers), ii) Managers and policy makers (micro-, meso-, and macro levels), and iii) Technological expertise. Representativeness of different healthcare tiers (Primary care, Hospital care, Intermediate care, and Macro-management) was also considered.

The interviews addressed the following items relative to complex chronic patients: i) problems associated to the identification of such patients, ii) roles and interactions among healthcare professionals in the care of those patients, iii) satisfactoriness of the current management of complex chronic patients, iv) challenges, unmet actionable needs and proposals for management, v) proposals to reduce unplanned hospitalizations in complex chronic patients, and vi) challenges, unmet actionable needs and proposals to enhanced transitional care after hospital discharge.

All fifteen professionals participating in the interviews were invited to be active in the two design thinking sessions, but only nine of them accepted. The absences of the remaining six were due to scheduling conflict. Two additional leaders could attend only the design thinking sessions. Such that the final number of attendees to the two design thinking sessions was eleven, nine of them also participating in the interviews, as indicated in **Table 1S**.

Three blocks of additional information fed the debates: i) Statements of the regional government on deployment of integrated care [1] and on digital health strategy [28], ii) Results of the studies conducted in 2006 and 2015 [6,12] showing the efficacy-effectiveness gap alluded to above, and iii) A recent study conducted at Hospital Clinic Barcelona (HCB) [25], combining predictive modelling for mortality and early re-admissions after hospital discharge and a cluster analysis in 761 patients.

**Figure 2:**
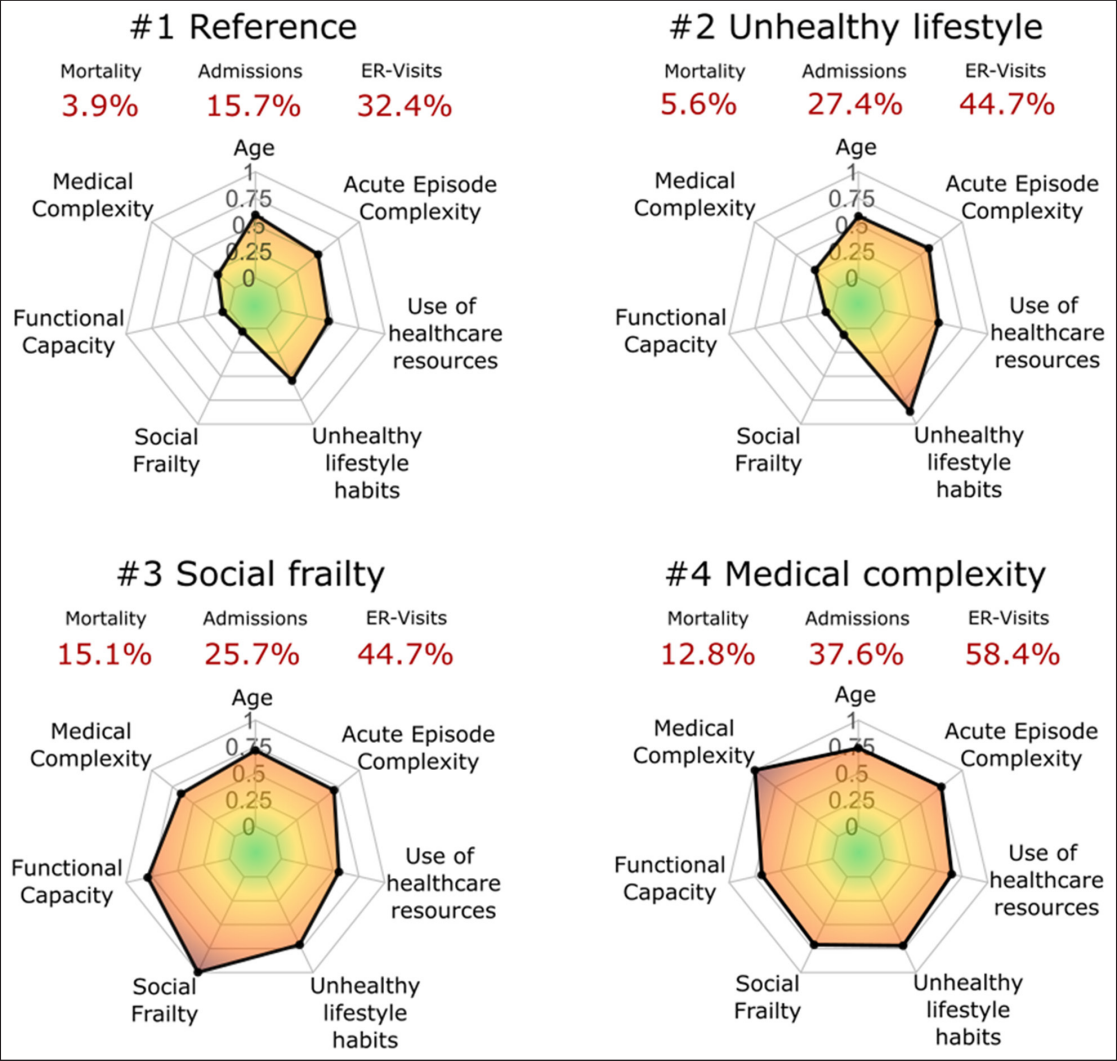
Radar plots of the main characteristics of the four clusters of patients identified in [25]. All the features are normalised and grouped into seven categories: i) age; ii) medical complexity; iii) functional capacity; iv) social frailty; v) unhealthy lifestyle habits; vi) use of healthcare resources; and vii) acute episode complexity. The mortality rates, hospital admissions and emergency room (ER) visits are displayed in red.

As mentioned, the first design thinking session was devoted to the analysis of the current regional management of complex chronic patients. The input information and the results of the interviews were processed to generate a Context Analysis **(Figure 1S)** and an Empathy Map. This map, divided into four quadrants, captured the perspectives of patients/professionals, detailing what they: said, did, thought and felt [29,30] **(Figure 2S)**. Discussion and consensus-building took place regarding the ideas presented, and proposals were collected. The results of the first design thinking session contributed to generate a SWOT analysis (identifying: strengths, weaknesses, opportunities, and threats) and to prepare a summary of the results of the session **(Figure 3S)**, that were used to feed the second session focused on exploring actions to improve complex chronic patients’ outcomes within a care continuum scenario. The Impact-Feasibility Matrix **(Figure 4S)**, generated from the debate, helped to assess the potential impact and effort required for each planned action. This analysis played a pivotal role to define recommendations for next steps.

The Confirmation Phase – The assessment of value generation and transferability of Hospital at Home in Catalonia (ES) during the period 2015–2019 [25,31], carried out in the context of JADECARE, offered the opportunity to analyse a large dataset of post-discharge episodes.

A total of 49,604 episodes from 27 different hospitals were examined. The analysis included patients’ characteristics, multimorbidity-complexity, use of healthcare resources and expenditure during four well-defined periods: i) one year before the acute episode, ii) during the acute episode requiring hospital admission, iii) 30-days, and iv) 90-days after hospital discharge. A large portion of the patients fell into the spectrum of complex chronic patients, as reported in [24], with no differences in health outcomes between modalities of hospitalization, either Hospital at Home or in-hospital admissions.

The report [24] combines a quantitative analysis of the results with a qualitative assessment of Home Hospitalization, and post-discharge episodes, carried out by a panel of seven experts. The panel included 1-to-2 representatives of the most relevant organizations in implementing or assessing Hospital at Home services in Catalonia: two members from the Catalan-Balearic Society of Hospital at Home, two staff members from the Catalan Health Service, one staff member from the Health Quality and Assessment Agency of Catalonia (AQuAS), and two experts from the local JADECARE team. Four out of the seven experts were clinical leaders of different Hospital at Home programs. A qualitative research and service design specialist was recruited to facilitate and lead the discussions of the expert panel.

The quantitative analysis of the hospital admission episodes, and the results of a survey previously administered to the panel of seven experts, fed the debates in the two subsequent design thinking sessions, held in November and December 2022, saw participation from by the same group of seven experts (Figure 1, **Table 2S**). It is of note that the analysis of heterogeneities among the 27 hospitals [24] in terms of traits and services outcomes was very useful to generate conclusions in the Consolidation phase.

### Setting the scene Main outcomes of the co-design process

The Discovery phase identified the following four actionable factors as main explanatory determinants of the efficacy-effectiveness gap:

1. *Change management* was found as the most relevant determinant requiring attention for action. However, several different factors/needs were considered under change management, namely: i) a better definition of the boundaries and interplay among different modalities of services depicted in Table 1, with emphasis on integration between health and social care services, ii) a definition of professionals’ profiles and roles required for enhanced management of selected chronic patients to be included in a program to prevent unplanned hospitalizations, iii) unmet specific needs in terms of education and training of those professionals, iv) request for a deeper debate defining patients’ profiles, taking into account their unique requirements for treatment and/or care, and v) the need to improved support for shared care agreements across different levels of healthcare and for collaborative work.
2. *Personalization of the interventions –* The results of the cluster analysis are depicted in Figure 2, reproduced from [23]. The Figure displays the main traits of the four clusters of patients identified in the analysis of 761 hospital discharges at HCB, as well as their mortality rate and use of healthcare resources after discharge. Clusters #1 and #2 include patients with a mean age in the early seventies. It is of note that patients in cluster #2, with potentially reversible risk factors related to unhealthy lifestyles, clearly showed worse health status and poorer prognosis than those in cluster #1. Whereas individuals in clusters #3 and #4 showed a similar mean age in the mid-eighties, with high medical or social complexity, respectively. These results fostered a lively debate on service selection depending on the patient profile, for example, home care or intermediate care programs (Clusters #3 and #4) versus community-based prevention of unplanned hospitalizations (Clusters #1 and #2).
  The results of the cluster analysis [25,26] reinforced the need for exploring the boundaries and interplay among different modalities of clinical and social care services indicated in Table 1, as well as the debate on cure and/or care needs in specific groups of complex chronic patients. Moreover, the personalization in terms of the type of concurrent diseases, and disease severity, focuses on the complementarities between disease-focused *vs* patient-oriented management. Also, case management strategies to monitor the progress of the patient’s status should be considered. These aspects highlight the importance of carefully selecting services and dynamically personalizing interventions over time. Moreover, such requirements have also a significant impact on the roles of the professionals involved in the preventive program.
  The debate suggested the need for exploring the potential of predictive modelling to feed clinical decision support tools that could contribute to personalization of transitional care action plans at hospital discharge. The ultimate goal is to improve the efficiency of care integration between hospital and community services, ensuring a seamless transition for patients.
3. *Mature Digitalization –* Current digital support was considered not to meet the requirements for the management of complex chronic patients. The group identified the following requirements to provide scalable digitalization: i) support collaborative work with an adaptive case management approach. By implementing such management modality, integrated care processes can be designed, developed, and implemented in a way that empowers patients, clinicians, and other stakeholders, leading to improved health outcomes and better patient experiences., ii) supporting cloud-based digital health tools with an adaptive case management approach, interoperable (ad-hoc) with existing electronic health records from different suppliers across healthcare tiers.
4. *Adoption and assessment –* The transition from evidence-based efficacy of any intervention to the demonstration of effectiveness and healthcare value generation assessed using a Triple or Quadruple Aim approach [32] was considered a must. Also, evaluation of equity in terms of service accessibility, Quintuple Aim [33], was considered necessary. Nevertheless, the applicability of the current assessment tools in real-world scenarios shows major limitations. As reported in [34] there is an urgent need to develop and validate applicable tools to evaluate Patient Reported Outcomes/Patient Reported Experiences (PROMs/PREMs), or their surrogates, for mainstream interventions in real-world scenarios. Also, identifying relevant key performance indicators (KPI) and elaborating appropriate dashboards for quality assurance after service adoption were proposed as core requirements. To this end, refinement of the comprehensive evaluation approach described in [35], aiming at enhancing its applicability, was strongly recommended.

The Discovery phase supported the hypothesis that an efficient program [26] to prevent unplanned hospitalizations in selected complex chronic patients could be designed [26] by properly articulating the four areas described above. Moreover, the analysis of the heterogeneities among the 27 healthcare providers [26], including the assessment of the post-discharge period, carried out the Consolidation phase was confirmatory of the considerations raised in the Discovery phase. The two design thinking sessions of the Confirmation phase indicated a high potential of the Hospital at Home teams to foster productive interactions, between hospital-based professionals and community-based teams, for reengineering transitional care after hospital discharge.

## Next steps

The research team used the recommendations generated in the two-phase qualitative analysis to define the foundations for a program aiming at preventing unplanned hospitalizations in selected complex chronic patients, described as follows.

The program’s main aims of are: i) facilitate early detection and management of exacerbations, as well as early identification of undesirable events after hospital discharge, ii) patients’ empowerment for effective self-management, and iii) shared care agreements and collaborative work across different care levels.

The profile of targeted candidates for the program should fulfil the following traits: i) multimorbid outpatients at high risk for hospital admissions because of a previous history of repeated severe exacerbations requiring admissions, ii) patients with a high morbidity burden, measured with the Adjusted Morbidity Groups [36,37] score, that is, all those cases allocated in the at-risk stratum of the population risk pyramid of Catalonia (i.e. above the 80^th^ percentile of the regional health risk distribution) and iii) patients not included in the home care or intermediate care programs depicted in Table 1.

The Adjusted Morbidity Groups score is an aggregative index based on diagnostic codes, which indicates the burden of an individual’s morbid conditions through a disease-specific weighting deduced from statistical analysis based on mortality and the utilization of health services. Moreover, all hospital post-discharges with a health risk level above percentile 80 should be evaluated as potential candidates for personalized 30-days transitional care interventions.

The research team proposes three sequential phases to finetune and evaluate the program to prevent unplanned hospitalizations, facilitating its adoption as a mainstream service at the end of a two-year period (2024–2025), including: i) Preliminary Studies, ii) Assessment of preventive interventions, and iii) Adoption as a mainstream service.

During 2024–2025, testing will be conducted on a cohort of 200 multimorbid outpatients, selected on two primary criteria: i) multimorbid individual allocated close to the peak of the Catalan risk stratification pyramid (> P_80_) [36,37], and ii) those diagnosed with chronic obstructive pulmonary disorders of moderate to severe intensity as one of the main diagnoses. Such a cohort will be followed up for a longer period, as part of the EU project, “Knowledge for improving indoor air quality and health” (K-Health in Air) [38]. The project‘s goal is to investigate the relationships between indoor air quality on health outcomes of chronic patients.

Table 2 indicates the main actions, directly derived from the co-creation process, to be evaluated and subsequently incorporated into the mainstream program to prevent unplanned admissions in selected complex chronic patients.

**Table 2:** Actions to be included in the program for prevention of unplanned hospitalizations.


ACTIONS	COMMENTS

**1. CHANGE MANAGEMENT**	

1.1. Define flexible clinical processes with a holistic approach.	Design the framework of the clinical process considering: i) clinical endpoints, actions associated to main diagnosis and co-morbidities, as well as environmental and social factors.

1.2. Define roles and profile of the advanced care nurse	Key roles are: i) patients’ empowerment for self-management, ii) care and cure actions following plans defined in 1.1, iii) early detection/management of exacerbations. Double ascription to primary care & Hospital at Home teams. Coordination with intermediate care service modalities

1.3. Integrate primary to quaternary preventions	Evolution from current focus on primary prevention to integration of all prevention levels in the management of the program candidates

1.4. Redefine interactions between nurse and patient	Initial motivational intervention followed by patient’s agreements on a personalized care plan (non-pharmacological interventions). Patient’s activation and empowerment following the procedures reported in [32,33]

1.5. Training programs for professionals and patients	Education/training of professionals and patients before the program initiation following the innovative approaches reported.

**2. PERSONALIZATION OF THE INTERVENTIONS**	

2.1 Service selection	Selection of the patient as candidate to the program or allocation into other service modalities indicated in Table 1

2.2. Harmonize disease- vs patient-oriented approaches.	The intervention (personalization of the clinical process) must consider the individual diseases (type, severity, and progress), as well as their potential interactions (also regarding pharmacological aspects).

2.3. Consider social and environmental factors	The holistic approach of the intervention requires consideration of the non-clinical aspects (social status, education level, environment, etc…) that may have influence on health status

2.4. Consider evolution of health status overtime	The characteristics of the intervention decided in the initial evaluation requires adaptation to the progress of the patient’s condition. The balance between cure and care should be considered.

**3. MATURE DIGITAL SUPPORT**	

3.1. Adaptive Case Management (ACM)	Combinations of multiple factors influencing the patient’s health status, and unexpected events (exacerbations), requires flexible management of the clinical process which can be achieved with an ACM approach.

3.2. Communication Channel	A communication channel based on chat (including intelligent chatbots) with multimedia support is essential to provide proactive interactions among stakeholders.

3.3. Collaborative work	Digital support to collaborative work among professionals and with patients across healthcare tiers in a must for executing share care agreements.

3.4. Capture of patient’s self-tracking data	Efficient patients’ input data from: i) non-disruptive sensors, ii) chat with the advanced care nurse, and/or iii) short questionnaires (with Likert scales) may play a relevant role in decision-making and knowledge generation.

3.5. Ad-hoc integration	Cloud-based technologies with ad-hoc integration with providers’ health information systems is the proposed approach for provision of digital support to the service.

**4. APPLICABLE ASSESSMENT IN REAL-WORLD SETTINGS**	

4.1. Evaluation of the process of implementation	Evolution of classical tools for assessment of the service deployment (i.e. Consolidated Framework for Implementation Research, CFIR [34] must be adopted to enhance applicability in real-world settings.

4.2. (PROMS)/(PREMS)	Role of sensor measurements (i.e., Heart Rate Variability), info from the chat and short questions (Likert scale) are candidates to substitute classical questionnaires [33,35,36,37].

4.3. User-profiled dashboards	Identification of Key Performance Indicators (KPI) and build-up management dashboards can contribute to service quality assurance over time.


The initial six months, from January to June 2024, will be devoted to the evaluation of innovative strategies for patient assessment consisting of three parallel independent study protocols addressing early detection and enhanced management of acute episodes of exacerbations. The three areas to be explored are, respectively: i) feasibility and impact of continuously monitoring the heart rate variability, as compared with the periodic administration of standard health questionnaires (diaries) [39], ii) the feasibility of pragmatically capturing PROMS/PREMS using mobile technology [21,40,41,42]; and iii) the role of forced oscillation technique, as compared to forced spirometry (FS) for lung function assessment [43].

The subsequent phase will consist of personalized preventive interventions, following the recommendations indicated in Table 2. The interventions will be run by a nurse case manager over twelve months, from July 2024 to June 2025. The nurse will have a double assignment to the primary care teams and to the research team at HCB. The digital support to the intervention will be based on a customized version of the cloud-based adaptive case management platform named Health Circuit™ [21]. Evaluation of the effectiveness and value generation of the intervention using a Quintuple Aim approach is planned, as well as the assessment of the deployment process with a pragmatic modification of the Consolidated Framework for Implementation Research (CFIR) approach [44]. The identification of Key Performance Indicators (KPI), and elaboration of a dashboard, for subsequent continuous quality assurance and management of the intervention, will be made.

The final six months, July to December 2025, will be used for refining the profiles of the target candidates and adjusting the program’s characteristics to meet the requirements for mainstream service adoption. This period will also be employed to consolidate the final steps of the certification process for the digital tools supporting the service.

## Discussion

The integrated care case practice took advantage of the lessons learned throughout the regional adoption of digitally supported integrated care guided by the two Catalan Health Plans 2011–2015 and 2016–2020 [19,20].

JADECARE (2020–2023) [10] has provided an adequate frame for critically evaluating the achievements and failures over more than one decade of adoption. Also, has prompted several quantitative assessments [25,45] that have contributed to the qualitative analysis. To our understanding, the current study introduces innovative practices that, after assessment in real world settings, can contribute to the global discourse on integrated care for chronic patients.

The Discovery phase allowed a structured analysis of the unmet needs of the regional management of complex chronic patients and facilitated the identification of four actionable areas for improvement, tackling: i) organizational aspects and novel professional roles, ii) personalization of the services, iii) innovations in digital support fostering collaborative work and use of the adaptive case management principle, and iv) structured support to the assessment of the transition from a study protocol to a sustainable adoption of the intervention. It is of note that the outcomes of the Confirmation phase were fully aligned with those previously identified in the Discovery phase, confirming the role of Hospital at Home as a relevant facilitator of vertical integration.

Beyond the recommendations alluded to above, the integrated care case practice has activated the application of specific technological and organizational solutions, generating synergies with early phases of the EU project K-Health-in-Air [38] as a testing field of the program for the prevention of unplanned hospitalizations during 2024–2025. It is reasonably expected that, at the end of the two-year period, the finetuned interventions will be ready for adoption as a mainstream service in a real-world scenario.

The authors are cognizant of the fact that certain intricacies of the organizational solutions presented in the current integrated care case practice may need site-specific adaptations when contemplating their applicability to other European scenarios that exhibit varying health system structures.

To our knowledge, the current study illustrates all the steps of the long journey from the necessary demonstration of evidence-based efficacy with randomized controlled trials, to a sustainable adoption of the intervention, that is: i) need for assessment of effectiveness, ii) evaluation of the deployment process using implementation science methodologies, iii) assessment of healthcare value generation, and iv) identification of KPI (and elaboration of user-profiled dashboards) for continuous quality assurance of the service after adoption. Since those steps are expected for most integrated care interventions, we believe that the recommendations raised by the current study are generalizable to other use cases beyond the program for preventing unplanned hospitalizations.

## Lessons Learned

The two-phase co-creation process has generated the following key learnings:

1. **A nurse case manager in the community**, can play a central role for enhanced management of complex chronic patients, also bridging with hospital-based specialists, as well as with other modalities of clinical and social care services for chronic patients.
2. **Personalization of the interventions** aiming at preventing unplanned hospitalizations according to: i) the evolving patient’s health status, ii) the relative weight of the different patient’s morbidities, as well as iii) to other determinants of frailty and complexity, constitutes a key requirement.
3. **Innovative cloud-based digital support**, following adaptive case management principles, and interoperable with different health and social care suppliers, fosters more informed decisions through collaborative work and facilitates end-used engagement.
4. **The underwent co-creation process has demonstrated its effectiveness** as a systematic and robust pathway for identifying the key elements necessary for the intervention’s transition from demonstrating evidence-based efficacy to being adopted as a value-based mainstream service. Those steps, reported in the integrated care case practice, are generalizable to other integrated care services.
5. **Transferability of recommendations, for the four actionable areas, at European level is possible**. But the need for site specific organizational adjustments shall be considered according to the characteristics of each health system.

## Conclusions

The present integrated care case practice describes the co-design process carried out to identify four crucial actionable factors (change management, personalization of the interventions, advanced digitalization and improved real-world service evaluation) influencing the observed efficacy-effectiveness gap in previous studies that aimed to test interventions for preventing unplanned hospitalizations in complex chronic patients.

Based on those main determinants, the study provides recommendations for a scalable program for preventing unplanned hospitalizations targeting selected complex chronic patients and transitional care strategies after hospital discharge. Moreover, the research proposes the sequential steps to pave the way towards the adoption of the intervention as a mainstream value-based service.

## Additional File

The additional file for this article can be found as follows:

10.5334/ijic.7724.s1 Online supplementary material. Tables 1S and 2S and Figures 1S- 4S.

## Figures and Tables

**Table:** 

AQUAS:	Agency for Health Quality and Assessment of Catalonia
CAPSBE:	Consortium of primary health care Barcelona Esquerra
CCP:	Complex chronic patients
EEG:	Efficacy-effectiveness gap
ESCA:	Catalonia health survey
HCB:	Hospital Clinic of Barcelona
JADECARE:	Joint Action on implementation of digitally enabled integrated person-centred care
KPI:	Key performance indicators
PROMs:	Patient reported outcomes measures
PREMs:	Patient reported experience measures
RCT:	Randomized controlled trial
WHO:	World Health Organization
