# Supplementary material for: Toward Sustainable Adoption of Integrated Care for Prevention of Unplanned Hospitalizations: A Qualitative Analysis
Source: Int J Integr Care. 2024 Jun 28;24(2):28. doi: 10.5334/ijic.7724 (PMC11212778; doi:10.5334/ijic.7724)
Supplement: Online supplementary material. — Tables 1S and 2S and Figures 1S- 4S. [file ijic-24-2-7724-s1.pdf]

Toward Sustainable Adoption of Integrated Care for Prevention of Unplanned Hospitalizations: a qualitative analysis

Herranz C. et al. 2023

Online supplementary material

For the two phases of the study, Discovery and Consolidation, the interviews were always conducted by two persons: the first author (Carme Herranz) and a qualitative research and service design specialist (Fernando Ozores, Buenaidea, http://www.buenaidea.es/). At the time of the study, Carme Herranz was a practicing nurse, and pre-doctoral student, with more than five years of experience in clinical research.

The interviews had a mean duration of 45 minutes each, whereas the mean duration of each design thinking session was 120 min. There were no previous personal relationships between researchers and the participants in the qualitative analysis, either patients or professionals. No bias was detected, except for the common interest of professionals and researchers for the research topic. The characteristics of the professionals participating in the design thinking sessions are depicted in **Tables 1S** (Discovery) and **2S** (Consolidation) wherein names, expertise & position, filiations, and level of participation are reported.

The selection of the 5 patients interviewed was based on the inclusion of most characteristic profiles seen in the clinical practice, whereas the main criterion for the selection of the 24 professionals participating in the two phases of the study was their representativeness, at different levels/disciplines, in the Catalan Health System. Their agendas were the only factor explaining the dropouts of some professionals in the design thinking sessions of the Discovery phase.

The patients' interviews were done at home or at the clinics depending upon availability and convenience, whereas the encounters with professionals were done using teleconference. All design thinking sessions were also carried out through teleconference.

Guidance was provided to the participants in the interviews through well-defined scripts, as indicated in the main manuscript. Moreover, the design thinking sessions were guided using the background material indicated in the main manuscript as well as the maps and summary reports depicted in **Figures 1S-4S**. No repetition of interviews was needed. The design thinking sessions were recorded, and summary reports were generated thereafter.

Codes, subthemes, and themes were generated using Atlas.ti 9 software.

Table 1S – Participants in the Discovery Phase: interviews and/or design thinking sessions.

Name	Expertise & Position	Filiation	Participation
Zoe Herreras	General Practitioner (GP) & Head of Care Processes	CAPSBE	I only
Antoni Sisó	GP & CAMFIC president	CAPSBE	I + DT
Susanna Torres	Social worker	CAPSBE	I only
Luis González	Nurse & Technician	CAPSBE	I + DT
Juan José Zamora	Nurse & Head of nursing processes	ICS	I + DT
Esther Limón	GP & Education coordinator & CAMFIC treasurer	ICS	I+DT
Gemma Yago	Advanced practice nurse at the Diabetes Unit	HCB	I+DT
David Font	MD & Strategy and Planning Director	HCB	I+ DT
Jose Antonio Rodríguez	Nurse & Coordinator Geriatric Unit	HCB	I only
Carmen Hernandez	Nurse & Innovation Unit	HCB	I only
Joan Escarrabill	MD & Director of Chronic Care	HCB	I+DT
Marco Inzitari	MD & Director of Intermediate Care	Parc Sanitari Pere Virgili	I + DT
Jordi Piera	Computer Engineer & Director of the Digital Health Strategy Office. CatSalut	Ministry of Health	I+ DT
Joan Carles Contel	Nurse & Staff member of the Chronic Care Program	Ministry of Health	I only
Oscar Solans	MD & Functional manager of eHealth	CatSalut	I only
Jordi Ambàs	Director of the Integrated Care Agency	Ministry of Health	DT only
Belen Enfedaque	Deputy director of primary and community care	Ministry of Health	DT only

GP: General Practitioner; CAPSBE: Primary Care Consortium at Barcelona-Esquerra; I: Interview; CAMFIC: Catalan Society of Primary Care; DT: Design Thinking sessions; ICS: Institut Catalan of Health; MD: Medical Doctor; CatSalut: Catalan Health Services is the single-public payer.

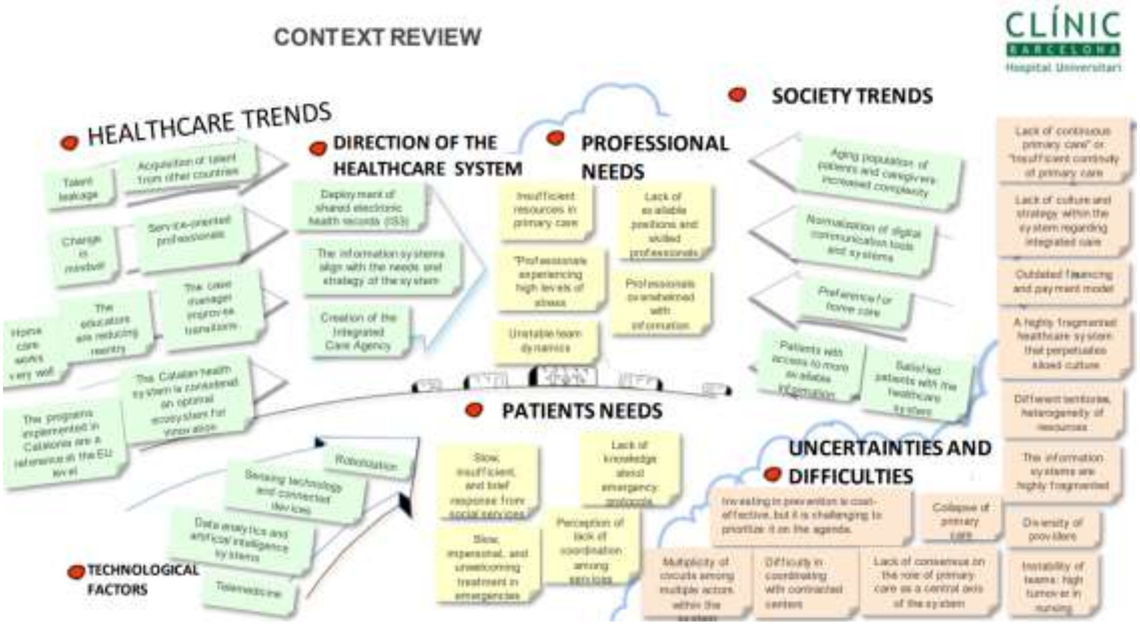

Figure 1S. Context Analysis*. General understanding of the patient's and experts' ideas collected during the interviews, aligned with seven different topics related to healthcare implementation.*

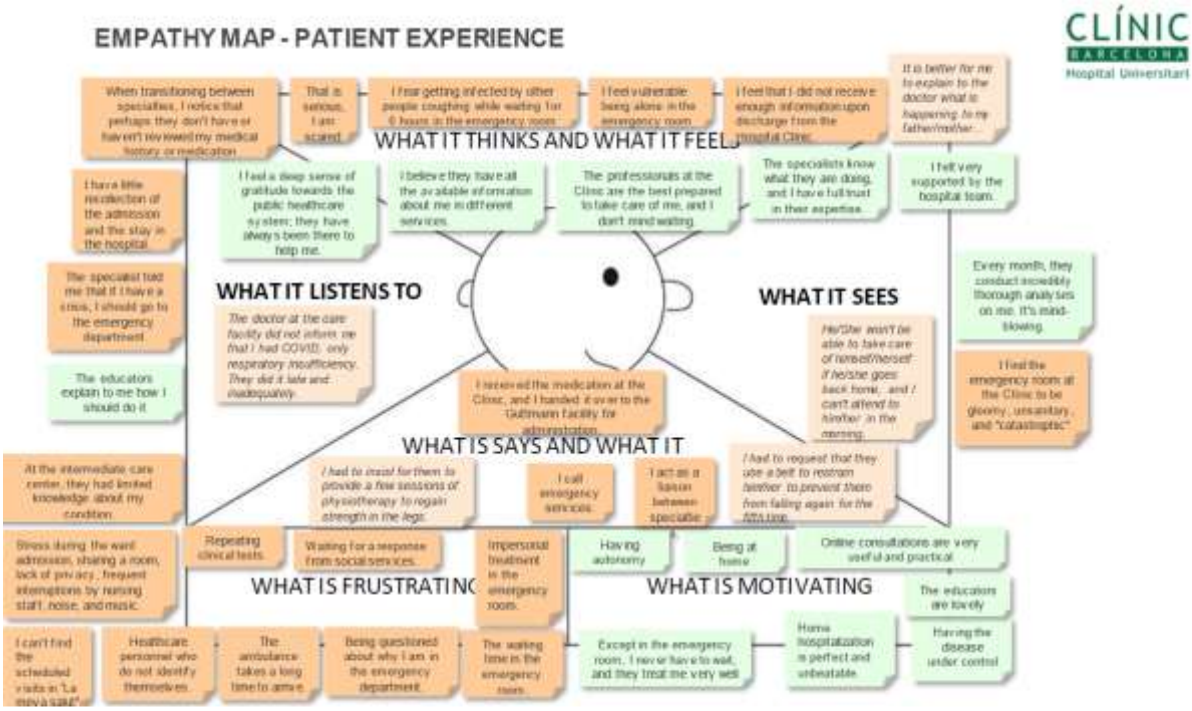

Figure 2S*. **Empathy Map**. Summary of the patients' interviews. Focused on their experience in the care process, ranging from the onset of the episode to the stay and discharge in intermediate care. Four areas that are confined to patient perception, segregated by whether they are discerned as a frustrating or motivating situation.*

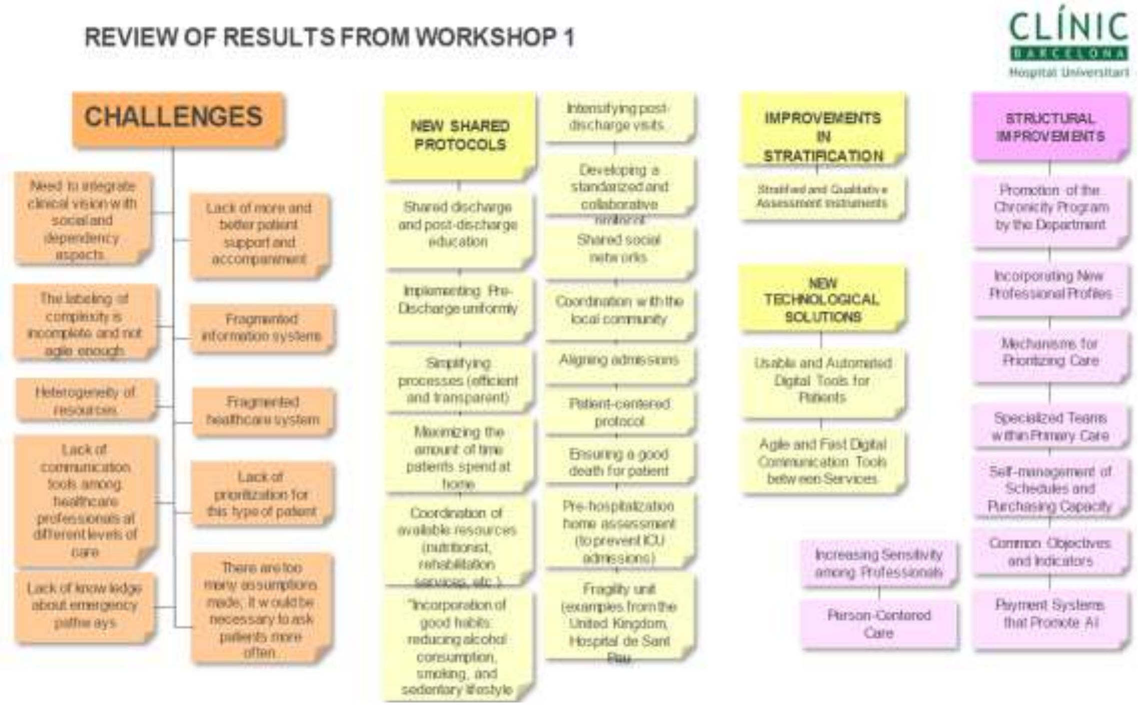

Figure 3S. Results from Workshop 1*. Key challenges and proposals gathered during the first DT design session were most relevant to the participants.*

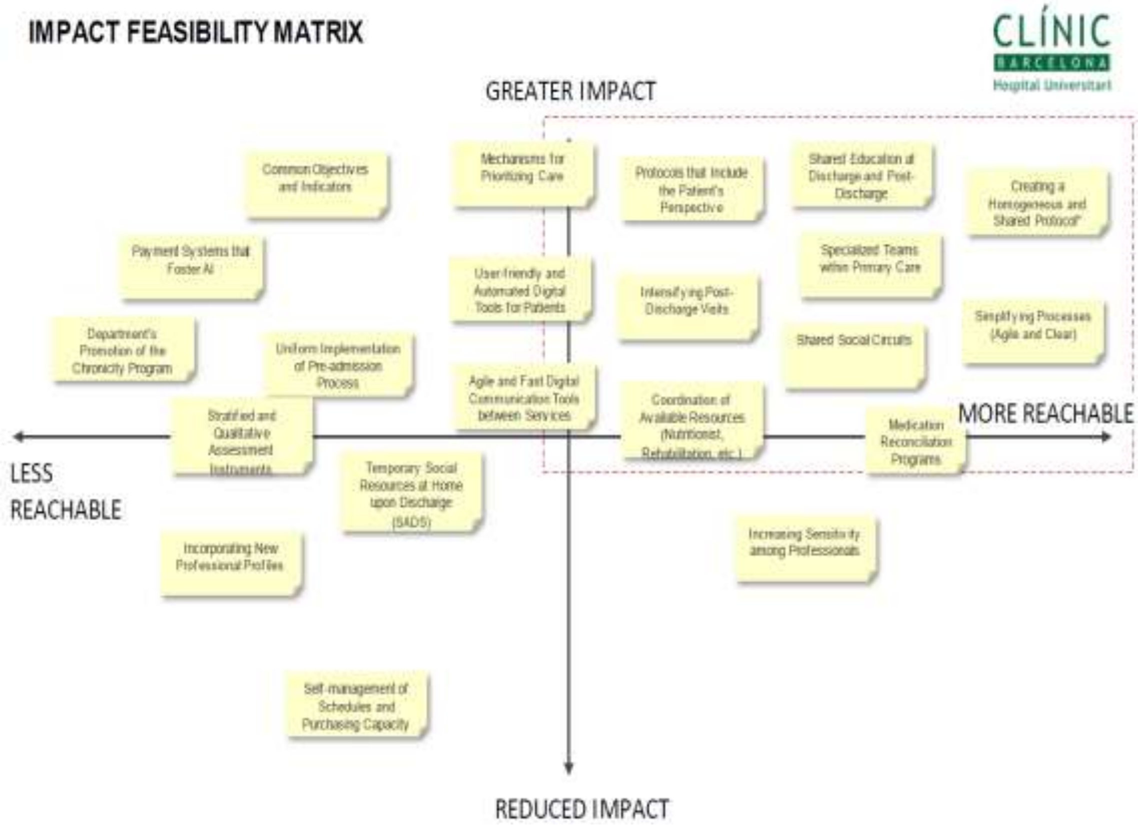

Figure 4S. Impact feasibility matrix. *This illustration was generated during the second DT session with the most important variables detected. A discussion was held regarding the operational aspects of the proposed solutions. The Impact feasibility matrix was enhanced for better understanding and making informed decisions, allowing us to identify areas of*

advantage, areas that need improvement, potential opportunities to leverage, and potential outcomes to mitigate.

CONFIRMATION PHASE

Table 2S. Participants in the Confirmation phase: Survey (S) and Design Thinking (DT) sessions

Name	Expertise & Position	Filiation	Participation
David Nicolas	MD & Coordinator of the HaH	HCB	S + DT
Francesc Xavier Jiménez	MD & Coordinator of the HaH	Hosp. Vall d'Hebron	S + DT
Eulalia Villegas-Bruguera	MD & Coordinator of the HaH	Hosp. Dos de Maig	S + DT
Carme Hernandez	Nurse. PhD & Innovation Unit & Former HaH coordinator	HCB	S + DT
Mireia Espallargues	Staff member (HaH specialist)	AQuAS	S + DT
Montserrat Suárez	Staff member (HaH specialist)	CatSalut	S+ DT
Elvira Torné	Staff member (HaH specialist)	CatSalut	S + DT

MD: Medical Doctor; HaH: Hospital at Home, HCB: Hospital Clinic de Barcelona; S: Survey; DT: Two Design Thinking sessions; AQuAS: Catalan Health Quality Agency; CatSalut: Catalan Health Services is the single-public payer.
